# Draft Genome Assembly of Filamentous Brackish Cyanobacterium *Limnoraphis robusta* Strain CS-951

**DOI:** 10.1128/genomeA.00846-15

**Published:** 2015-09-03

**Authors:** Anusuya Willis, Matthew Parks, Michele A. Burford

**Affiliations:** a Australian Rivers Institute and School of Environment, Griffith University, Nathan, QLD, Australia; b Environmental Futures Research Institute, Griffith University, Nathan, QLD, Australia

## Abstract

*Limnoraphis robusta* CS-951 is a sheathed, filamentous benthic, nonheterocystous cyanobacterium. It was isolated from brackish water and identified morphologically as *Lyngbya majuscula*. We report the draft genome of *L. robusta* CS-951, with a genome size of 7,314,117 bp, a 41.6% GC content, and 6,791 putative protein-coding genes assembled into 361contigs.

## GENOME ANNOUNCEMENT

Benthic filamentous cyanobacteria occur globally in freshwater, brackish, and marine systems. Eutrophication can stimulate large blooms that can have significant negative environmental, economic, and social impacts by smothering seagrass beds and producing toxins (1, 2). Recent molecular taxonomic reevaluation of the widely identified problem species *Lyngbya majuscula* revealed multiple polyphyletic genera (3, 4). These genera circumscribe a continuum of morphologically similar cyanobacteria ranging from freshwater to marine, benthic to planktonic, diazotrophic, and nondiazotrophic taxa, including *Okeania*, *Trichodesmium*, *Lyngbya*, *Moorea*, and *Oscillatoria* (5, 6).

A nonaxenic benthic estuarine cyanobacteria strain, CS-951 (CCAP 1446/4, Norfolk, United Kingdom), characterized by sheathed unbranched filaments and first identified morphologically as *Lyngbya majuscula*, was supplied by the Australian National Algae Culture Collection (CSIRO, Australia) and cultured in 50% seawater f/2 medium (7) at 23°C under 15 µmol photons m^2^ s^−1^ with a 12 h/12 h light-dark cycle.

DNA was isolated from culture using a phenol-chloroform method (8) following mechanical agitation with silica/zirconium beads in cetyltrimethylammonium bromide (CTAB) buffer, and treatment with proteinase K and lysozyme. A genomic library was prepared following Nextera XT protocol (Illumina, Inc.) and sequenced on an Illumina MiSeq platform (Ramaciotti Centre, Australia) using 150-bp paired-end sequencing. Initial *de novo* assemblies were performed with Velvet v.1.2.03 (9), under default parameters, with Kmer lengths of 75, 85, and 95, and insert sizes estimated by mapping reads to scaffolds from the *L. majuscula* 3L assembly (10) and a 5.8-kbp cistron containing nitrogen fixation (*nif*) genes (GenBank accession no. DQ078751.1) (BWA v.0.5.9 [11] and SAMtools v.0.1.18 [12]). Initial contigs were evaluated based on the number of contigs assembled, *N*_50_, maximum contig size, and total length of assembled contigs. *De novo* assemblies were refined using kmer lengths of 85 and expected coverage depths of 7 and 20. Additional assemblies were performed using MaSuRCA v.2.3.2 (13) and A5 v.20150518 (14) under default parameters. Contigs from contaminating heterotrophic bacteria were removed from assemblies based on GC content, phylogenetic affinity, and coverage depth. Lastly, qualifying contigs from the three assemblies were merged into final contigs using CISA v.1.3 (15). Final assembled contigs were submitted to IMG ER and RAST for automatic annotation. The resulting draft genome for *L. robusta* CS-951 had 361 contigs covering 7,314,117 bp, with 41.6% GC content and 6,791 putative protein-coding genes.

The CS-951 16S rRNA gene sequence is indistinguishable from the planktonic freshwater *L. robusta* strain CCALA 996 (16). Positive identification of the nifHDK operon suggests CS-951 is nonheterocystous diazotrophic. BLAST searches employed to identify homology to cyanobacterial toxins with known genes (e.g., *LxtABCD, sxt*), returned no positive hits. Secondary metabolite and toxin biosynthesis genes were predicted by antiSMASH (17) using NRPS and/or PKS gene identification. Four predicted structures had no similarity to characterized toxins and represent novel secondary metabolites.

Benthic cyanobacteria are known for their production of diverse secondary metabolites, which may be species specific (5, 6). The availability of this genome allows for greater understanding of secondary metabolites.

### Nucleotide sequence accession numbers.

This whole-genome shotgun project has been deposited at DDBJ/EMBL/GenBank under the accession number LATL00000000. The version described in this paper is version LATL02000000.
